# MicroRNA-126 inhibits tumor proliferation and angiogenesis of hepatocellular carcinoma by down-regulating EGFL7 expression

**DOI:** 10.18632/oncotarget.11877

**Published:** 2016-09-06

**Authors:** Ming-Hua Hu, Chen-Yang Ma, Xiao-Ming Wang, Chen-Dong Ye, Guang-Xian Zhang, Lin Chen, Jin-Guo Wang

**Affiliations:** ^1^ Department of Surgery, Yijishan Hospital, Wannan Medical College, Wuhu 241001, P.R. China; ^2^ Department of Surgery, The Second Affiliated Hospital, Wannan Medical College, Wuhu 241001, P.R. China

**Keywords:** MicroRNA-126, EGFL7, hepatocellular carcinoma, proliferation, angiogenesis

## Abstract

This study aims to explore the effects of microRNA-126 (miR-126) on tumor proliferation and angiogenesis of hepatocellular carcinoma (HCC) by targeting EGFL7. HCC tissues and adjacent normal tissues were obtained from 71 HCC patients. Immunohistochemistry (IHC) was conducted to detect expressions of EGFL7 and VEGF and the micro-vessel density (MVD). HCC cell lines were collected and assigned into the blank, miR-126 mimics, miR-126 inhibitors, miR-126 mimics negative control (NC), miR-126 inhibitors NC, si-EGFL7, and miR-126 inhibitors + si-EGFL7 groups. Expressions of miR-126 and *EGFL7* mRNA were detected by qRT-PCR assay. The protein expressions of EGFL7 and VEGF were measured by Western blotting. MTT assay was used to measure the proliferation of HCC cells. Tumor xenograft model in nude mice was utilized to evaluate the influence of miR-126 on tumor growth. HCC tissues had higher miR-126 expression and lower *EGFL7* mRNA expression than adjacent normal tissues. Compared with the blank, miR-126 mimic NC, miR-126 inhibitor NC and miR-126 inhibitors + si-EGFL7 groups, the protein expressions of EGFL7 and VEGF and cell proliferation were reduced in the miR-126 mimics and si-EGFL7 groups, while the opposite trend was found in the miR-126 inhibitors group. Compared with the blank and miR-126 inhibitors + siRNA-EGFL7 groups, tumor size, tumor weight, and MVD of transplanted tumors in nude mice were significantly reduced in the miR-126 mimics and siRNA-EGFL7 groups, while the opposite trend was found in the miR-126 inhibitors group. In conclusion, miR-126 could inhibit tumor proliferation and angiogenesis of HCC by down-regulating EGFL7 expression.

## INTRODUCTION

Fast growth, high degrees of malignancy and metastasis, and high recurrence rate after surgical resection lead to poor outcomes for hepatocellular carcinoma (HCC) patients [1]. HCC morbidity and mortality rank 5^th^ and 3^rd^, respectively, among malignant tumors worldwide, with an estimated 750,000 new cases and 700,000 deaths in 2008 [2, 3]. HCC is triggered by inactivation of tumor suppressor genes and abnormal activation of proto-oncogenes in liver cells, aberrantly regulating several signaling pathways that form complex molecular regulatory networks through “cross talk” to promote tumorigenesis [4]. Angiogenesis plays an important role in tumor formation, providing nutrients to tumor cells and promoting tumor growth and metastasis [5] Analyzing genes and pathways involved in HCC tumorigenesis and angiogenesis will reveal genetic changes important for improving HCC patient outcomes and enhancing diagnostic accuracy [6].

MicroRNAs (miRs), a class of highly conserved endogenous non-coding RNAs of about 20 to 25 nucleotides in length, can regulate target mRNA post-transcriptional expression or restrain translation [7]. MiR-126 is abundant in endothelial cells and can modulate blood vessel development and production through regulation of locally-expressed genes [8]. Also, miR-126 has been negatively associated with tumor occurrence, development and metastasis and appears to act as a tumor suppressor [9]. MiR-126 up-regulation was verified *in vitro* to inhibit breast cancer cell proliferation and invasion, indicating that miR-126 can act as a suppressor in breast cancer [10]. It has been found that epidermal growth factor like domain 7 (EGFL7) is over-expressed during pathophysiological angiogenesis [11], where it is secreted to the extracellular matrix, and triggers the vascular sprouting process [12]. EGFL7 functions in the formation as well as maintenance of endothelial integrity [13] and has capability of inhibiting endothelial cell adhesion molecules making blood vessels leaky [14]. In a study performed by Hansen et al., he advocates that the assumption of low miR-126 and high EGFL7 expressions being linked to an increased metastatic potential [15]. However, the regulatory relationship between miR-126 and EGFL had not yet been explored with respect to tumor angiogenesis. Thus, in the present study, we studied the effects of miR-126 on tumor proliferation and angiogenesis of HCC by targeting EGFL7.

## RESULTS

### Comparison of miR-126, EGFL7 mRNA and protein expressions between HCC tissues and adjacent normal tissues

**Figure 1:**
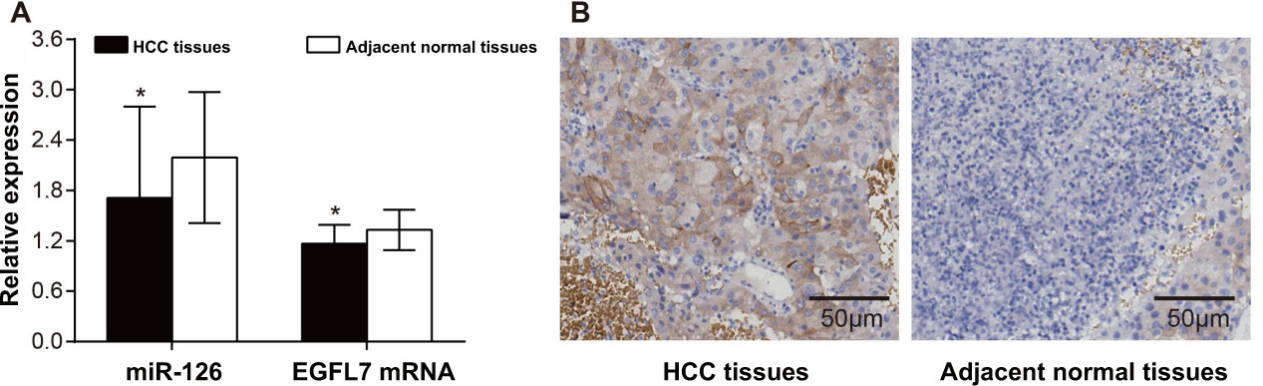
The expressions of miR-126, EGFL7 mRNA and protein in HCC tissues and adjacent normal tissues (**A**) The expressions of miR-126 and EGFL7 mRNA in HCC tissues and adjacent normal tissues detected by qRT-PCR. *P < 0.05 compared with adjacent normal tissues; (**B**) The expression of EGFL7 in HCC tissues and adjacent normal tissues detected by immunohistochemistry (× 200).

HCC tissues had higher miR-126 expression and lower *EGFL7* mRNA expression than adjacent normal tissues (both *P* < 0.05) (Figure 1A). The positive expression of EGFL7 protein showed brownish yellow to brown particles, mainly expressed in cytoplasm of HCC cells (Figure 1B). There was hardly positive expression of EGFL7 in the adjacent tissues. There were 64 cases (64/71, 90.14%) showing EGFL7 positive expression in HCC tissues, but only 19 cases (19/71, 26.76%) showing EGFL7 positive expression in adjacent normal tissues. A significant difference in the EGFL7 positive rate was found between HCC tissues and adjacent normal tissues (χ^2^ = 58.72, *P* < 0.05).

### Correlations of miR-126 and EGFL7 mRNA expressions with clinicopathological features of HCC

As shown in Table 1, miR-126 expression was negatively correlated with tumor size, liver cirrhosis and VEGF expression of HCC patients (all *P* < 0.05). However, miR-126 expression failed to be linked to age, gender, TNM stage, PVTT, capsular infiltration and HBsAg (all *P* > 0.05). Meanwhile, EGFL7 mRNA expression was positively correlated with tumor size, TNM stage, liver cirrhosis and VEGF expression of HCC patients (all *P* < 0.05) (all *P* < 0.05), but EGFL7 mRNA expression had no associations with age, gender, PVTT, capsular infiltration and HBsAg (all *P* > 0.05).

**Table 1:** Correlations of miR-126 and EGFL7 mRNA expressions with clinicopathological features of hepatocellular carcinoma

Feature	*n*	miR-126	*P*	EGFL7 mRNA	*P*
Age (years)			0.709		0.439
> 55	19	1.62 ± 1.42		1.06 ± 0.23	
≤ 55	52	1.74 ± 1.04		1.10 ± 0.21	
Gender			0.292		0.715
Male	57	1.78 ± 1.25		1.09 ± 0.23	
Female	14	1.41 ± 0.47		1.11 ± 0.17	
Tumor size			< 0.001		0.001
> 5 cm	51	1.40 ± 0.74		1.14 ± 0.20	
≤ 5 cm	20	2.49 ± 1.65		0.95 ± 0.22	
TNM stage			0.258		0.013
I-II stage	17	1.99 ± 0.94		0.98 ± 0.21	
III-IV stage	54	1.62 ± 1.22		1.13 ± 0.21	
Liver cirrhosis			0.014		0.023
Yes	53	1.51 ± 0.94		1.12 ± 0.23	
No	18	2.29 ± 1.57		0.99 ± 0.17	
PVTT			0.624		0.186
Yes	44	1.66 ± 1.19		1.12 ± 0.21	
No	27	1.80 ± 1.14		1.05 ± 0.15	
Capsular infiltration			0.782		0.164
Yes	38	1.67 ± 1.36		1.12 ± 0.24	
No	33	1.75 ± 0.91		1.05 ± 0.15	
HBsAg			0.661		0.124
Positive	63	1.69 ± 1.18		1.10 ± 0.21	
Negative	8	1.88 ± 1.13		0.98 ± 0.29	
VEGF			0.025		0.001
Positive	16	1.09 ± 0.54		1.26 ± 0.24	
Negative	55	1.86 ± 1.23		1.05 ± 0.20	

Note: miR-126, microRNA-126; EGFL7, epidermal growth factor-like domain 7; TNM, tumor nodes metastasis; PVTT, portal vein tumor thrombus; VEGF, vascular endothelial growth factor.

### Targeting relationship between miR-126 and EGFL7

**Figure 2:**
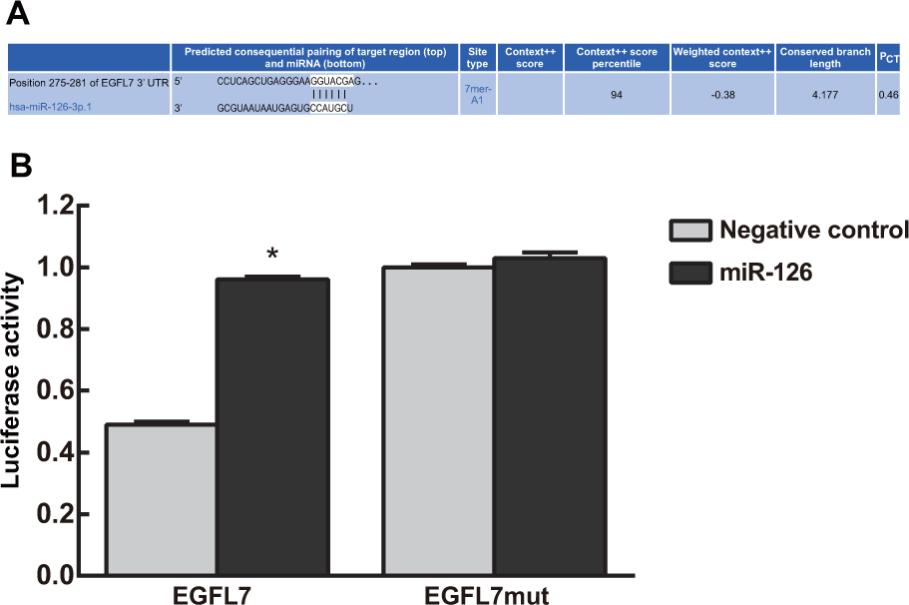
Targeting relationship between miR-126 and EGFL7 (**A**) The TargetScan database showed that EGFL7 was a potential miR-126 target gene; (**B**) Luciferase reporter assay indicated that miR-126 bound the EGFL7 3′UTR, directly inhibiting EGFL7 transcription. *P < 0.05 compared to the negative control group.

The TargetScan database showed that EGFL7 was a potential miR-126 target gene (Figure 2A). Luciferase reporter assay indicated that the EGFL7-3′-UTR luciferase signal dropped by 80% in the miR-126 mimics group (*P* < 0.05). However, the EGFL7 mut-3′-UTR luciferase signal was not reduced in any group (*P* > 0.05). These results implied that miR-126 bound the EGFL7 3′UTR, directly inhibiting EGFL7 transcription (Figure 2B).

### The expression of miR-126 in HCC cells after transfection

The expression of miR-126 in the miRNA-126 mimics group was the highest compared with other groups (*P* < 0.05) (Table 2). Compared to the blank, miR-126 inhibitor NC and si-EGFL7 groups, the expression of miR-126 was remarkably reduced in the miRNA-126 inhibitors and miR-126 inhibitors + si-EGFL7 groups (all *P* < 0.05). There was no difference in miR-126 expression between the blank, miR-126 mimic NC and miR-126 inhibitor NC groups (*P* > 0.05).

**Table 2:** The expression of miR-126 in SMMC-7721, MHCC-97H and HCCLM3 cells after transfection

Group	SMMC-7721	MHCC-97H	HCCLM3
Blank	1.00 ± 0.00	1.00 ± 0.00	1.00 ± 0.00
miR-126 mimics	2.85 ± 0.70^a^^b^^c^^d^	2.33 ± 0.58^a^^b^^c^^d^	2.02 ± 0.51^a^^b^^c^^d^
miR-126 inhibitors	0.50 ± 0.05^a^^b^^c^	0.66 ± 0.07^a^^b^^c^	0.74 ± 0.06^a^^b^^c^
miR-126 mimic NC	1.05 ± 0.20	1.06 ± 0.17	1.02 ± 0.16
miR-126 inhibitor NC	1.09 ± 0.12	1.03 ± 0.10	1.05 ± 0.13
si-EGFL7	1.06 ± 0.09	1.05 ± 0.11	1.01 ± 0.09
miR-126 inhibitors + si-EGFL7	0.52 ± 0.04^a^^b^^c^	0.69 ± 0.08^a^^b^^c^	0.78 ± 0.09^a^^b^^c^

Note: miR-126, microRNA-126; EGFL7, epidermal growth factor-like domain 7; NC, negative control;

a *P* < 0.05 compared with the blank group;

b *P* < 0.05 compared with the miR-126 mimic NC group

c *P* < 0.05 compared with the miR-126 inhibitor NC group

d *P* < 0.05 compared with the miR-126 inhibitors + si-EGFL7 group.

### The mRNA and protein expressions of EGFL7 in HCC cells after transfection

**Figure 3:**
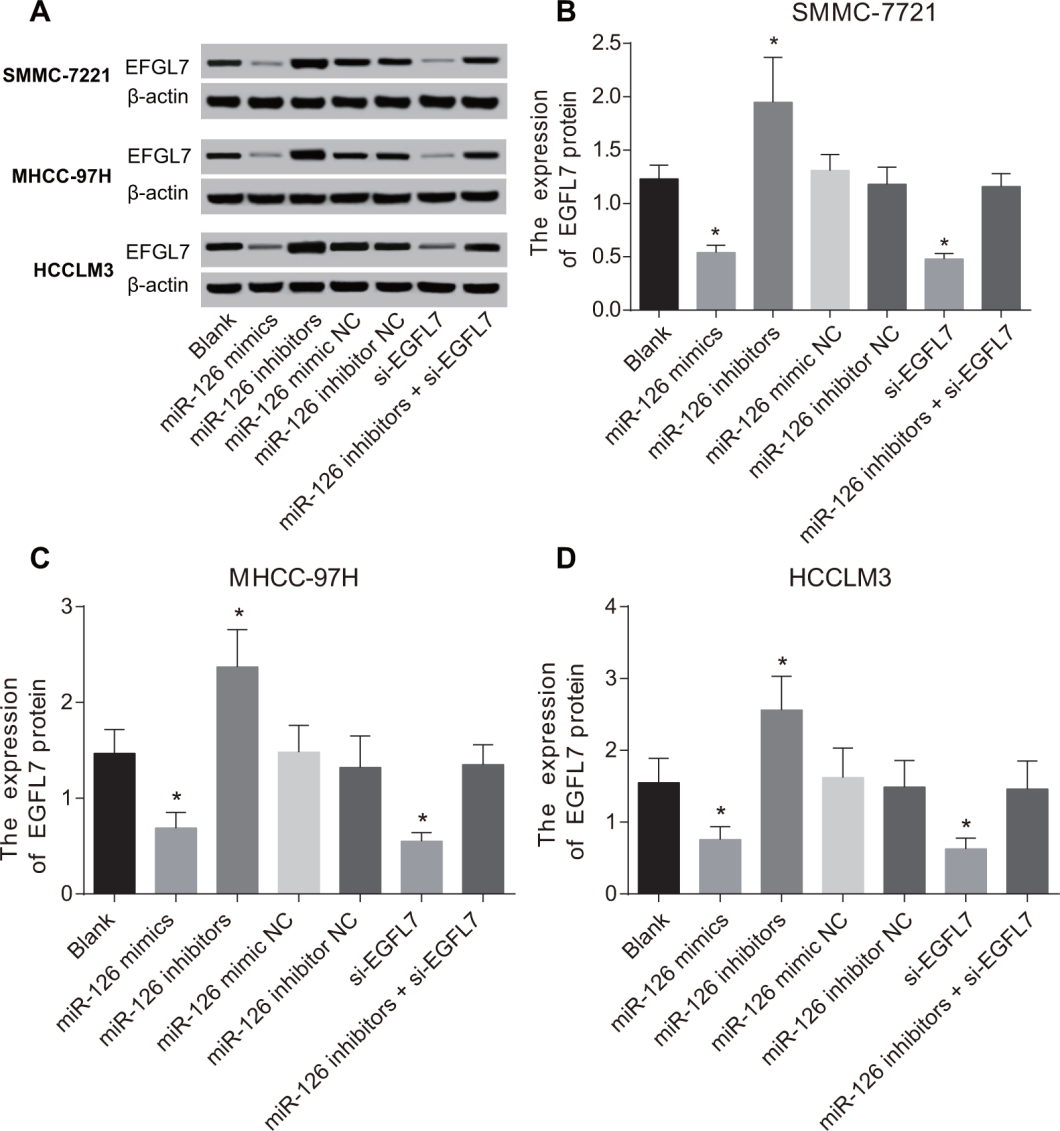
The protein expression of EGFL7 in SMMC-7721, MHCC-97H and HCCLM3 cells after transfection (**A**) The EFGL7 protein expression in each group detected by Western blotting; (**B**) Comparisons of the EFGL7 expression in SMMC-7721 cells among different groups; (**C**) Comparisons of the EFGL7 expression in MHCC-97H cells among different groups; (**D**) Comparisons of the EFGL7 expression in HCCLM3 cells among different groups. *P < 0.05 compared with the blank, miR-126 mimic NC, miR-126 inhibitor NC, or miR-126 inhibitor + si-EGFL7 groups.

Compared with the blank group, the mRNA and protein expressions of EGFL7 were unchanged in the miR-126 mimic NC, miR-126 inhibitor NC and miR-126 inhibitors + si-EGFL7 groups (*P* > 0.05). As shown in Table 3, the mRNA and protein expressions of EGFL7 were significantly reduced in the miR-126 mimics and si-EGFL7 groups (all *P* < 0.05). In the miR-126 inhibitors group, EGFL7 mRNA and protein expressions were increased (*P* < 0.05) (Figure 3).

**Table 3:** The mRNA expression of EGFL7 in SMMC-7721, MHCC-97H and HCCLM3 cells after transfection

Group	SMMC-7721	MHCC-97H	HCCLM3
Blank	1.00 ± 0.00	1.00 ± 0.00	1.00 ± 0.00
miR-126 mimics	0.57 ± 0.19^a^^b^^c^^d^	0.65 ± 0.20^a^^b^^c^^d^	0.70 ± 0.23^a^^b^^c^^d^
miR-126 inhibitors	1.23 ± 0.04^a^^b^^c^^d^	1.16 ± 0.07^a^^b^^c^^d^	1.14 ± 0.06^a^^b^^c^^d^
miR-126 mimic NC	0.98 ± 0.09	1.01 ± 0.08	0.99 ± 0.05
miR-126 inhibitor NC	1.03 ± 0.10	1.02 ± 0.07	1.02 ± 0.08
si-EGFL7	0.50 ± 0.17^a^^b^^c^^d^	0.58 ± 0.15^a^^b^^c^^d^	0.62 ± 0.21^a^^b^^c^^d^
miR-126 inhibitors + si-EGFL7	0.93 ± 0.11	0.95 ± 0.08	0.93 ± 0.09

Note: miR-126, microRNA-126; EGFL7, epidermal growth factor-like domain 7; NC, negative control;

a *P* < 0.05 compared with the blank group;

b *P* < 0.05 compared with the miR-126 mimic NC group;

c *P* < 0.05 compared with the miR-126 inhibitor NC group;

d *P* < 0.05 compared with the miR-126 inhibitors + si-EGFL7 group.

### Cell proliferation of HCC cells after transfection

**Figure 4:**
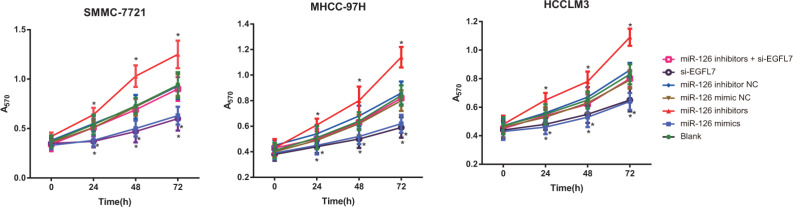
Cell proliferation of SMMC-7721, MHCC-97H and HCCLM3 cells after transfection among different groups detected by MTT assay *P < 0.05 compared with the blank, miR-126 mimic NC, miR-126 inhibitor NC, or miR-126 inhibitor + si-EGFL7 groups.

MTT assay results indicated that the proliferation of HCC cells in the blank, miR-126 mimic NC, miR-126 inhibitor NC and miR-126 inhibitors + si-EGFL7 groups exhibited no differences (all *P* > 0.05). At 24 h after transfection, compared with the blank, miR-126 inhibitor NC and miR-126 inhibitors + si-EGFL7 group, the proliferation of HCC cells was increased in the miR-126 inhibitors group and decreased in the miR-126 mimics and si-EGFL7 groups (all *P* < 0.05). There was no difference in the proliferation of HCC cells between the miR-126 mimics and si-EGFL7 groups (*P* > 0.05) (Figure 4).

### Influence of miR-126 on the expression of VEGF HCC cells after transfection

**Figure 5:**
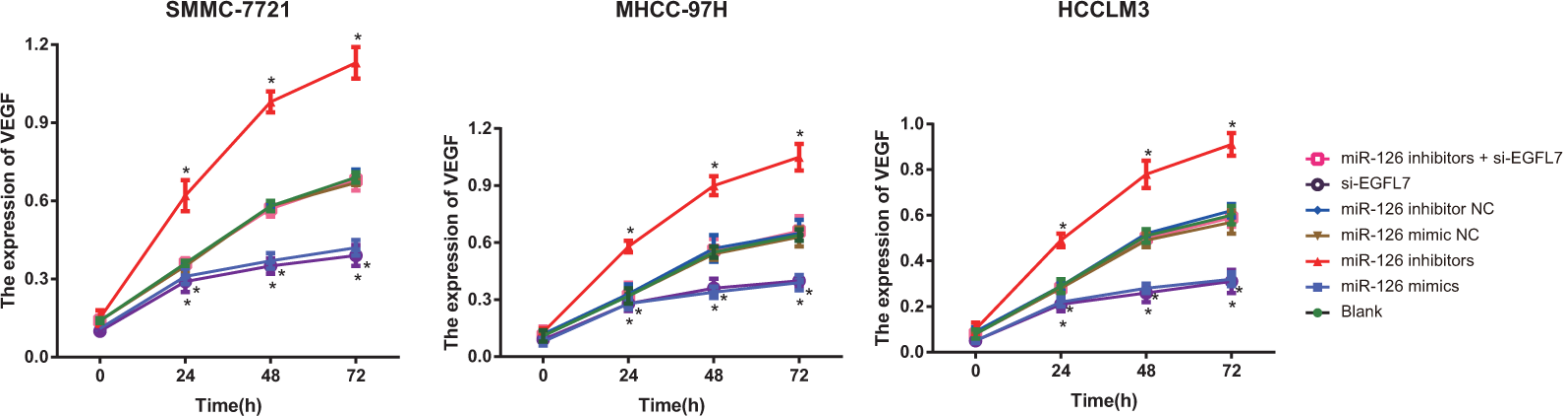
The VEGF expression of SMMC-7721, MHCC-97H and HCCLM3 cells after transfection among different groups detected by Western blotting *P < 0.05 compared with the blank, miR-126 mimic NC, miR-126 inhibitor NC, or miR-126 inhibitor + si-EGFL7 groups.

The expression of VEGF showed no difference in the blank, miR-126 mimic NC, miR-126 inhibitor NC, miR-126 inhibitors + si-EGFL7 groups in the same cell lines (all *P* > 0.05). There was also no difference in the expression of VEGF between the miR-126 mimics and si-EGFL7 groups (*P* > 0.05). At 24 h, 48 h and 72 h after transfection, the expression of VEGF was highest in the miR-126 inhibitors group and lowest in the miR-126 mimics and si-EGFL7 groups (*P* < 0.05) (Figure 5).

### Influence of miR-126 on tumor growth of transplanted tumors in nude mice

**Figure 6:**
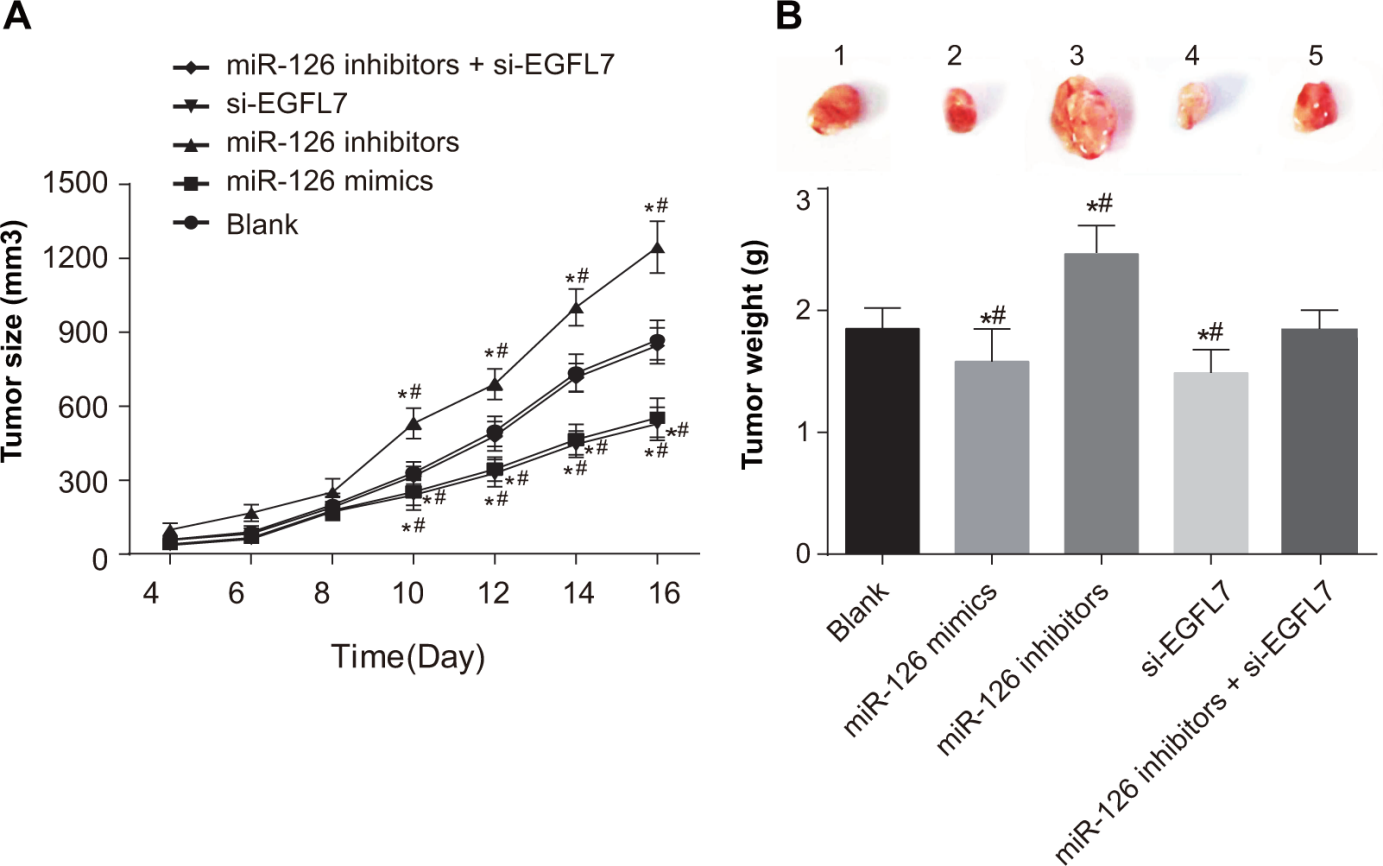
Tumor size and tumor weight of transplanted tumors in nude mice among different groups (**A**) Comparison of tumor size of transplanted tumors in nude mice among different groups; (**B**) Comparison of tumor weight of transplanted tumors in nude mice among different groups. *P < 0.05 compared with the blank group. **P < 0.01 compared with the miR-126 inhibitors + si-EGFL7 group.

All mice survived with a tumor formation rate of 100% and the transplanted tumors could be observed at 4 days after injection. Compared with the blank and miR-126 inhibitors + si-EGFL7 groups, significant increases in tumor sizes were observed in the miR-126 inhibitors group at the first 10 days after transfection (both *P* < 0.05), while the tumor size was decreased in the miR-126 mimics and si-EGFL7 groups (all *P* < 0.05) (Figure 6A). Tumor weights of transplanted tumors in the miR-126 inhibitor group were heavier than other four groups (all *P* < 0.05). Nevertheless, tumor weights of transplanted tumors in the miR-126 mimics and si-EGFL7 groups were lower than the blank and miR-126 inhibitors + si-EGFL7 groups (all *P* < 0.05) (Figure 6B).

### The mRNA and protein expressions of EGFL7 in transplanted tumors in nude mice

**Figure 7:**
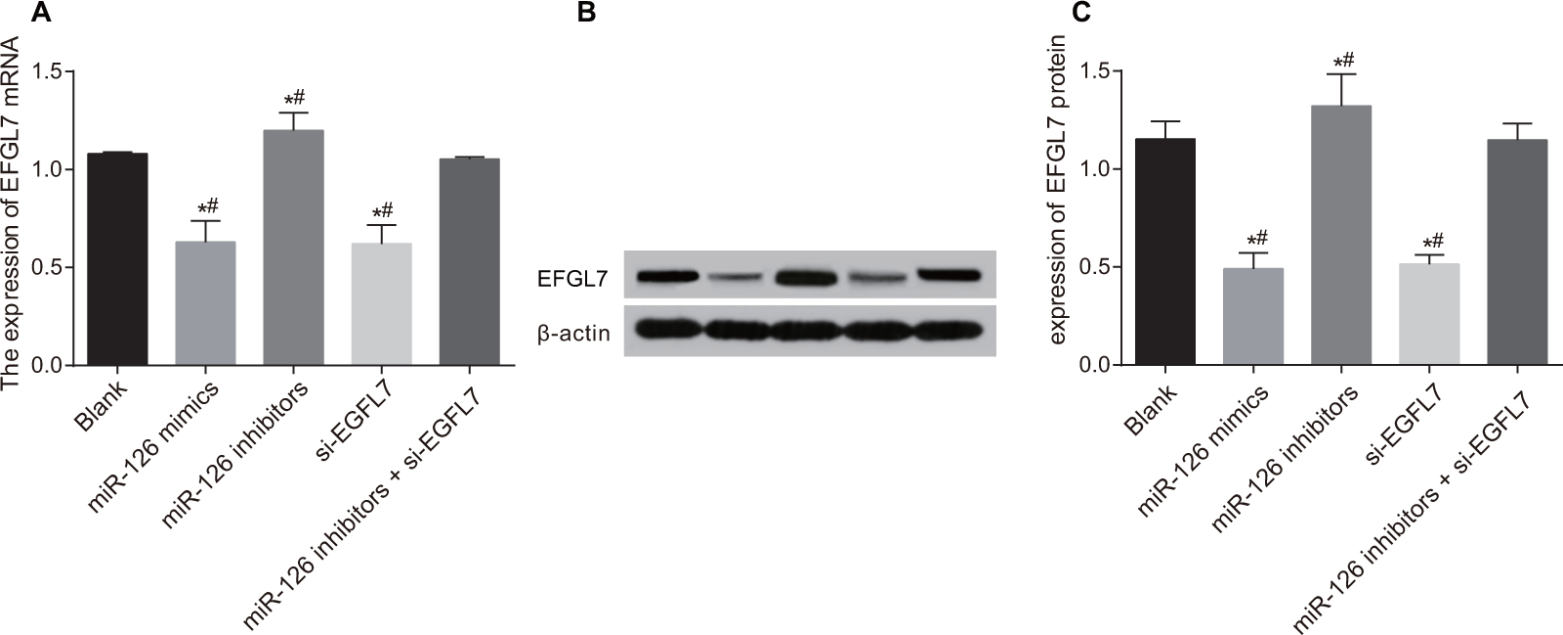
The mRNA and protein expressions of EGFL7 in transplanted tumors in nude mice among different groups (**A**) Comparison of the mRNA expression of EGFL7 in transplanted tumors in nude mice among different groups. (**B**) The protein expression of EGFL7 in transplanted tumors in nude mice among different groups detected Western blotting; (**C**) Comparison of the protein expression of EGFL7 in transplanted tumors in nude mice among different groups. *P < 0.05 compared with the blank group. **P < 0.01 compared with the miR-126 inhibitors + si-EGFL7 group.

The mRNA and protein expressions of EGFL7 were decreased in the miR-126 mimics and si-EGFL7 groups and increased in the miR-126 inhibitors group in comparison to the blank and miR-126 inhibitors + si-EGFL7 groups (all *P* < 0.05) (Figure 7).

### Serum ALT and VEGF levels in nude mice

As shown in Table 4, ELISA results showed that serum ALT and VEGF levels were declined in the miR-126 mimics and si-EGFL7 groups and elevated in the miR-126 inhibitors group compared to the blank and miR-126 inhibitors + si-EGFL7 groups (all *P* < 0.05). There was no difference in serum ALT and VEGF levels between the blank and miR-126 inhibitors + si-EGFL7 groups (both *P* > 0.05).

**Table 4:** Serum ALT and VEGF levels in nude mice among different groups

Group	ALT (U/L)	VEGF (pg/mL)
Blank	33.19 ± 3.86	55.15 ± 8.29
miR-126 mimics	15.50 ± 1.94*^#^	45.66 ± 5.07*^#^
miR-126 inhibitors	393.35 ± 24.01*^#^	81.43 ± 8.27*^#^
si-EGFL7	14.90 ± 2.02*^#^	43.87 ± 3.95*^#^
miR-126 inhibitors + si-EGFL7	35.02 ± 4.24	58.33 ± 6.41

Note: miR-126, microRNA-126; ALT, alanine aminotransferase; VEGF, vascular endothelial growth factor; EGFL7, epidermal growth factor-like domain 7;

* *P* < 0.05 compared with the blank group;

# *P* < 0.05 compared with the miR-126 inhibitors + si-EGFL7 group.

### The VEGF-positive rate and MVD of transplanted tumors in nude mice

**Figure 8:**
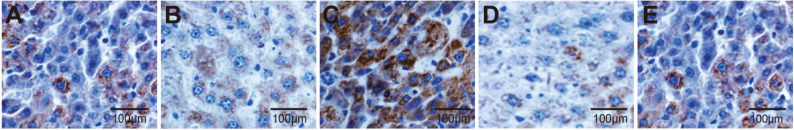
The VEGF-positive rate of transplanted tumors in nude mice among different groups detected by immunohistochemistry (× 200) (**A**) The blank group; (**B**) The miR-126 mimics group; (**C**) The miR-126 inhibitors group; (**D**) The si-EGFL7 group; (**E**) The miR-126 inhibitors + si-EGFL7 group.

The expression of VEGF in transplanted tumors was concentrated in the cell cytoplasm, with light yellow and brown staining (Figure 8). There were more VEGF-positive cells in the miR-126 inhibitors group than in the blank and miR-126 inhibitors + si-EGFL7 groups (both *P* < 0.05), and VEGF was only weakly positive in the miR-126 mimics and si-EGFL7 groups. The VEGF-positive rate in the miR-126 inhibitors group (66.7%) was higher than the blank (25.0%), miR-126 mimics (0%) and si-EGFL7 (8.3%) groups (all *P* < 0.05).

**Figure 9:**
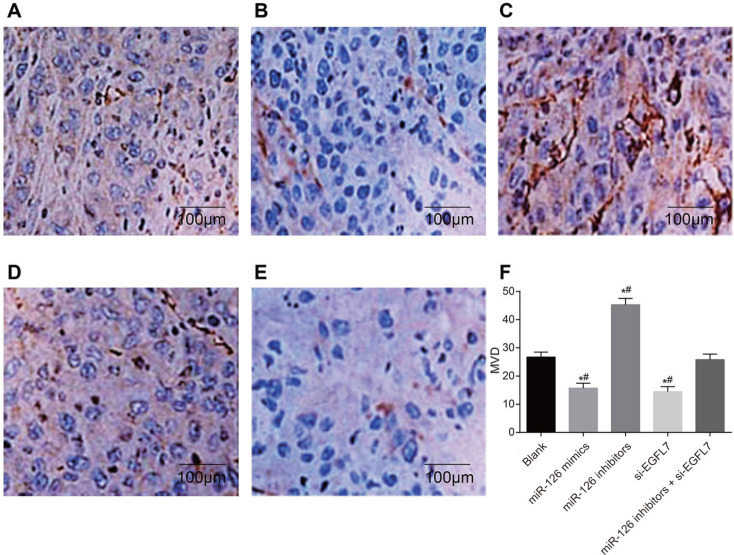
The number of CD31-positive micro-vessels of transplanted tumors in nude mice among different groups detected by immunohistochemistry (× 200) (**A**) The blank group; (**B**) The miR-126 mimics group; (**C**) The miR-126 inhibitors group; (**D**) The si-EGFL7 group; (**E**) The miR-126 inhibitors + si-EGFL7; (**F**) Comparison of the number of CD31-positive micro-vessels of transplanted tumors in nude mice among different groups. *P < 0.05 compared with the blank group. **P < 0.01 compared with the miR-126 inhibitors + si-EGFL7 group.

As shown in Table 5, the number of CD31-positive micro-vessels was highest in the miR-126 inhibitors group and lowest in the miR-126 mimics and si-EGFL7 groups compared to the blank and miR-126 inhibitors + si-EGFL7 groups (all *P* < 0.05). There was no difference in the number of CD31-positive micro-vessels between the blank and miR-126 inhibitors + si-EGFL7 groups (*P >* 0.05) (Figure 9).

**Table 5:** The VEGF-positive rate of transplanted tumors in nude mice among different groups

Group	VEGF-positive expression				
	–	+	++	+++	P
Blank	0	4	5	3	Ref.
miR-126 mimics	0	10	2	0	0.032
miR-126 inhibitors	0	0	4	8	0.041
si-EGFL7	0	10	1	1	0.044
miR-126 inhibitors + si-EGFL7	0	3	5	4	0.867

Note: miR-126, microRNA-126; VEGF, vascular endothelial growth factor; EGFL7, epidermal growth factor-like domain 7; Ref. reference.

## DISCUSSION

Recent evidence showed that down-regulation of miR-126 plays a pivotal role in various cancers, and ectopic miR-126 expression could suppress cell proliferation and growth in lung, gastric and breast cancers [9, 10, 16]. EGFL7 was subsequently found to be involved in cellular responses such as migration and angiogenesis, and was confirmed as a potential miR-126 target via bioinformatics analyses. Our study investigated the relationship between miR-126 and EGFL7, as well as the effects of miR-126 on tumor proliferation and angiogenesis of HCC. We found that miR-126 indeed targets EGFL7 and inhibits the proliferation of HCC cells. Thus, miR-126 may suppress tumor angiogenesis and reduce MVD.

Png, *et al.* reported that endogenous miR-126 non-cell-autonomously controls endothelial cell aggregation to metastatic breast cancer cells *in vitro* and *in vivo*. This indicated that miR-126 repressed recruitment of endothelial cells and metastatic angiogenesis by targeting the novel pro-angiogenic genes and metastasis biomarkers, *MERTK*, *IGFBP2* and *PITPNC1* [17]. Jiao, *et al*. reported miR-126 down-regulation in pancreatic ductal adenocarcinoma, with elevations in KRAS and CRK, and confirmed that miR-126 directly inhibits KRAS translation via interaction with a KRAS 3′UTR “seedless” site [18]. Sun, *et al.* showed that miR-126, through targeting EGFL7, may suppress A549 cell proliferation *in vitro*, and inhibit tumor growth *in vivo* [9]. Chen et al. revealed that decreased expression of miR-126 is associated with metastatic recurrence of HCC [19]. Consistent with previous studies, our results indicated that down-regulation of miR-126 was detected in the HCC tissues and associated with tumor size and liver cirrhosis. We may assume that down-regulation of miR-126 may contribute to tumorigenesis and tumor progression. Our study found that low miR-126 expression was associated with tumor progression via VEGF and EGFL7 activation. Our work employed the dual luciferase reporter gene assay, using the TargetScan database to identify likely miR-126 target genes. Importantly, our study provided evidence that miR-126 can bind to the EGFL7 3′UTR and inhibit transcription, indicating that miR-126 regulates EGFL7. Fish, *et al*. reported that miR-126 mediates EGFL7 expression in a negative feedback loop to control angiogenic signaling and vascular integrity [20]. Consequently, we performed cell transfection experiments to investigate the targeting relationship between miR-126 and EGFL7 to understand its function in HCC. The results showed that when EGFL7 levels decreased, miR-126 expression increased, suggesting that higher miR-126 levels would inhibit cell proliferation and VEGF expression. We also validated *in vivo* that miR-126 down-regulation increased serum ALT and VEGF levels, and enhanced micro-vessel density in tumors.

Our studies confirmed that tumor angiogenesis is important for HCC cell growth, invasion and metastasis. Importantly, EGFL7, as a type of epidermal growth factor, is highly expressed in the blood vessels of a variety of tumor and tumor adjacent tissues [21]. Moreover, EGFL7 may play an essential role in promoting blood vessel formation and maintenance of vascular integrity via VEGF activation. miR-126 knockdown during zebrafish and mouse embryogenesis reportedly delayed angiogenic sprouting, collapsed blood vessels, and caused widespread hemorrhages and partial embryonic lethality [20]. Thus, miR-126, through EGFL7, may indirectly reduce VEGF expression and blood vessel formation as an intracellular inhibitor of angiogenic signaling [22, 23]. Confirming this, we found that miR-126 was negatively correlated with EGFL7 and VEGF expressions in HCC tissues.

In conclusion, our study clearly illustrated the targeting relationship between miR-126 and EGFL7 in HCC. Furthermore, miR-126 could inhibit tumor proliferation and angiogenesis of HCC by down-regulating EGFL7 expression. Therefore, miR-126 and EGFL7 could serve as potential therapeutic targets for HCC.

## MATERIALS AND METHODS

### Subjects

From January 2014 to January 2016, HCC tissues and corresponding adjacent normal tissues (more than 2 cm from tumor margin and no cancer cell confirmed by pathological examination) were collected from 71 HCC patients at the Department of Surgery of Yijishan Hospital, Wannan Medical College, including 57 male and 14 female with a mean age of 49.14 ± 9.07 years (ranged from 18 to 65 years). These specimens were handled by RNA preservation solution, stored at −80°C. All patients were confirmed as HCC by pathological examination after operation, and there were 63 HBsAg-positive patients s and 8 HBsAg-negative patients. The tumor diameter ranged from 0.5 cm to 18.5 cm. There were 53 cases with liver cirrhosis and 44 cases with portal vein tumor thrombus (PVTT). In accordance with tumor node metastasis (TNM) staging system of Union for International Cancer Control (UICC) and American Joint Committee on Cancer (AJCC) [24], there were 17 cases in in stage I/II and 54 cases in stage III/IV. This study was approved by the Ethical Committee of Yijishan Hospital, Wannan Medical College. Written informed consents were obtained from all study subjects.

### Immunohistochemistry (IHC) for the detection of EGHL7 expression

Streptavidin-biotin-peroxidase-complex (SABC) method was employed to performing immuno histochemistry analysis, using goat-anti-human EGFL7 polyclonal antibody (1:50, Santa Cruz Biotechnology, Inc, CA, USA). Rabbit-anti-goat second antibody kit (Maixin Biotech. Co., Ltd, Fuzhou, China) was operated according to manufacturer's instructions. Using ethylene diamine tetraacetic acid (EDTA) buffer (0.01 M, pH = 6.0), the specimens was heated in a microwave oven for 15 min for antigen thermal repair. Blocking solution of rabbit serum instead of primary antibody was used as a negative control. The staining results were read and scored by professional physician with optical microscope (Olympus Corporation, Tokyo, Japan). Staining determination and scoring followed Shimizu method [25]. Staining intensity scoring: cytoplasm without color, 0 point; cytoplasm with pale yellow, presenting nebulous shape, 1 point; cytoplasm with yellow granular, 2 points; cytoplasm with uniform dark yellow, 3 points. Positive cell proportion scoring: positive cell numbers ≤ 10%, 0 point; 10%–40%, 1 point; 40%–70%, 2 points; ≥ 70%, 3 points. When two kinds of scores were added, 0–1 point was defined as negative and more than 2 points as positive.

### Cell culture

HCC SMMC-7721, MHCC-97H, and HCCLM3 cells were obtained from the Cell Bank of the Shanghai Institutes for Biological Sciences, Chinese Academy of Sciences. 50,000 cells/mL were inoculated into 6-well plates and maintained in RPMI 1640 medium containing 10% fetal bovine serum (FBS), 100 U/mL penicillin and 100 mg/L streptomycin, in a humidified atmosphere of 5% CO_2_ at 37°C. Cells were passaged using 0.125% trypsin containing 0.1% ethylenediamine tetraacetic acid (EDTA). Cells were prepared for use at the logarithmic growth phase.

### Cell transfection and grouping

Human SMMC-7721, MHCC-97H, and HCCLM3 cells at the logarithmic growth phase were collected and assigned into 7 groups:: (1) blank group (no transfection with any sequence); (2) miR-126 mimics group (transfection with miR-126 mimics); (3) miR-126 mimic NC group (transfection with miR-126 mimics negative control plasmid); (4) miR-126 inhibitors group (transfection with miR-126 inhibitors); (5) miR-126 inhibitor NC group (transfection with miR-126 inhibitors negative control plasmid); (6) si-EGFL7 group (transfection with si-EGFL7); and (7) miR-126 inhibitors + si-EGFL7 group (transfection with miR-126 inhibitors and si-EGFL7). All of the above transfection sequences were synthesized by GenePharma (Shanghai, China). Cells at the logarithmic growth phase were seeded in 24-well plates at 5 × 10^5^ cells/well. After 24 h, when converged to 70%, the cells were subjected to transfection in Hiperfect transfection kit (QIAGEN, Germany) according to the instructions. Before transfection, the cells were placed into fresh and antibody-free RPMI 1640 containing 10% FBS. With addition of Hiperfect transfection reagent into RPMI 1640, transfection complex was formed and was added into the plate. The plate was subjected to cell incubation for 6 h, and culture medium was changed. After 24 h of transfection, total RNA was extracted from cells and subjected to fluorescence quantitative PCR, and miRNA-126 and EGFL7 levels were measured. After 72 h, total protein was extracted and EGFL7 expression was detected by western blotting.

### Luciferase reporter assays

Potential miR-126 target genes were collected via TargetScan.org. SMMC-7721 cells at logarithmic growth phase were inoculated into 96-well plates (4,000 cells/well, 100 ul/well) and cultivated for 24 h. The EGFL7 gene 3′UTR (GCTCTAGACCTGCAAGAAAGACTCGTGA) was sequenced via polymerase chain reaction (PCR), and the PCR product was inserted downstream of the Luciferase gene in the pmirGLO plasmid (Promega, Beijing, China). MiR-126 and target gene binding sites were predicted based on bioinformatics analyses of TargetScan for site-directed mutagenesis. Namely, Luciferase reporter gene carrier including wide-type EGFL7 3′UTR and mutant-type EGFL7 3′UTR was constructed, and then EGFL7 3′UTR (both wide-type and mutant-type) two sides were inserted with Xho1I and HindIII. The pRL-TK vector (TaKaRa Biotechnology Co., Ltd., Dalian, China) expressing renilla luciferase was used as an internal reference to control for differences in cell number and transfection ratio. miR-126 and miR negative control (miR-NC) were separately transfected into SMMC-7721 cells with Lipofectamine^TM^2000 (Life Technologies, USA). Dual luciferase activity was determined according to the manufacturer's instructions (Promega, Beijing, China).

### MTT assay

Cell proliferation was assessed using the Cell Proliferation Reagent Kit I (Roche, Basel, Switzerland). Transfected cells were inoculated into 96-well plates at three wells per group according to the instructions. MTT (10 ul, 5 mg/ml) was added to the reaction system. After 4 h, 100ul methyl-sulfoxide was added and reacted for 30 min at 37°C. Cell activity was detected by measuring absorbance at 570nm using a Thermo Scientific Microplate Reader (Thermo Fisher, USA). All experiments were conducted in quadruplicate and were repeated three times.

### Quantitative real time fluorescent polymerase chain reaction (qRT-PCR)

Total RNA was extracted from the HCC tissues and adjacent normal tissues, transfected cells and excised transplanted tumor tissues using the RNeasy Mini Kit (QIAGEN, Germany) according to the manufacturer's instructions. A260/A280 ratio was measured and RNA concentration was determined via nanodrop ultraviolet spectrophotometer. RNA was reverse transcribed into cDNA using the RT Kit (Promega Corporation, USA). The Fluorescence quantitative PCR kit was purchased from GenePharma Co., Ltd., Shanghai, China. An ABI 7500 real-time fluorescence quantitative PCR instrument (Applied Biosystem, USA) was used with the following reaction (20 ul) conditions: 40 cycles at 95°C for 3 min, 95°C for 12 s and 62°C for 50 s. U6 was used as an internal reference for miR-126 mRNA, with β-actin for EGFL7 mRNA. PCR primers are listed in Table 6. Relative expression was calculated as 2^−ΔΔCT^ (ΔCt = Ct_miR- 126_–Ct_U6_, ΔΔCt = ΔCt_experiment group_–ΔCt_control group_).

**Table 6:** The primers for quantitative real time fluorescent polymerase chain reaction (qRT-PCR)

Gene	Sequence
miR-126	F: 5′-CAGTGCGTGTCGTGGAGT-3′
	R: 5′-GGGGCGTACCGTGAGTAAT-3′
U6	F: 5′-CGCTTCACGAATTTGCGTGTCAT-3′
	R: 5′-GCTTCGGCAGCACATATACTAAAAT-3′
EGFL7	F: 5′-AAAATGGAAGCCCCTCAACT-3′
	R: 5′-CAGGAGAGGGCAGACTAAGC-3′
β-actin	F: 5′-CATGTACGTTGCTATCCAGGC-3′
	R: 5′-CTCCTTAATGTCACGCACGAT-3′

Note: miR-126, microRNA-126; EGFL7, epidermal growth factor-like domain 7.

### Western blotting

Transfected cells and prepared tissues were washed with PBS, reacted in 100 ul of cell lysis buffer and incubated at 4°C for 30 min. After centrifugation at 12,000 g for 10 min, supernatant was collected and protein was concentrated using the Bradford method. Sodium dodecyl sulfate-polyacrylamide gel electrophoresis (SDS-PAGE, 12%) was performed with 50ug protein/lane, followed by protein transfer to polyvinylidene difluoride membranes. Membranes were blocked with 5% non-fat milk for 1 hour at room temperature. Mouse anti-human EGFL7, VEGF and GAPDH monoclonal antibodies (1:200) were separately applied to membranes at 4°C overnight. Goat anti-mouse IgG marked by IRDyeTM800DX (1:10,000) was then added to washed membranes. All antibodies were purchased from Upstate Corporation, USA. After incubation in the dark at room temperature for 1 hour, membranes were again washed and scanned using an infrared laser imaging system. Relative expression for each band was calculated as the ratio of the target band IOD to that of the corresponding internal reference (β-actin).

### Tumor xenograft model in nude mice

Sixty 5-week-old male BALB/c mice were purchased from Beijing HFK Bioscience Co., Ltd and were raised in a specific pathogen free (SPF) environment at room temperature (ranging from 22 to 26°C), with relative humidity ranging from 40 to 60%. All water, padding and fodder were sterilized at high temperatures and cages were sterilized every 3 d. After adaptive breeding for 12 d, 60 mice were divided into 5 groups (12 mice per group): the blank, miR-126 mimics, miR-126 inhibitor, si-EGFL7, miR-126 inhibitors + si-EGFL7 groups. The posterior flank of each mouse was injected with 0.1 ml suspended cell fluid. Tumor size was examined every 2 d and calculated as length × width^2^ × 0.5. Sixteen days later, anaesthetized mice were dissected for tumor tissue. This study was conducted in strict accordance with the recommendations in the Guide for the Care and Use of Laboratory Animals of the National Institutes of Health

### Enzyme-linked immunosorbent assay (ELISA)

Mice were subjected to blood draws from a main abdominal artery during tumor resection. Whole blood was collected in vacuous anticoagulant tubes and centrifuged at 1000 × g at room temperature for 5 h. Serum (supernatant) was quickly transferred into a −20°C refrigerator. A 50 ul volume of serum was used to detect ALT using the Hitachi 7600 automatic biochemical analyzer (Hitachi, Japan). Reagents were purchased from KOFA Biotechnology Co., Ltd. Guangzhou, China. 100 ul serum was used to evaluate VEGF expression via the Rat VEGF ELISA kit (Merck Millipore, German). The Thermo multifunctional microplate reader (Thermo Fisher, USA) was used for detection.

### Immunohistochemistry (IHC) for the detection of VEGF and micro-vessel density (MVD)

Immunohistochemistry was used to detect VEGF in mice tumor tissues. Tumor MVD was measured with CD31 as the positive index. Rabbit anti-human polyclonal antibodies VEGF, CD31 (Upstate, USA) and the EnVisionTM detection kit (DAKO, Denmark) were used for staining. Paraffin-embedded sections (3 um) were deparaffinized, hydrated, incubated at room temperature for 10 min and then washed in PBS for 10 min. After microwave antigen repair, sections were reacted in non-immune animal serum at room temperature for 10 min. Following addition of the first antibody (1:200), the section was placed in a wet incubator box at 37°C for 60 min. Sections were washed in PBS for 10 min, incubated using the EnVisionTM kit for 10 min, washed again in PBS for 10 min, incubated in the substrate-chromogen solution for 10 min and washed in distilled water. Sections then underwent DAB coloring, hematoxylin counterstaining and mounting with neutral gum. The primary antibody was replaced with PBS in negative controls. Under the microscope, we selected five different visual areas under high power, with a total of 100 cells. VEGF-positive cells presented granular yellow. Cell positivity was scored as follows: <5% positive cells = 0; 5 ∼15% = 1; 15∼50% = 2; > 50% = 3. Staining intensity was scored as follows: positive cells were uncolored = 0; yellow = 1; brownish yellow = 2; brown = 3. Final scores were calculated as the percentage of positive cells multiplied by staining intensity score: negative (−): 0–2; weakly positive (+): 3–5; positive (++): 6–8; strongly positive (+++): 9–12. CD31-positive cells in tumor tissues presented brown or brownish yellow and represented endothelial cells and tumor neovascularization. We identified “hot spots” (cancer nest peripheral vascular dense area) via light microscopy at 40 × magnification. The number of blood vessels in four different regions under 400 × magnification was also assessed. An area was considered microvascular when the lumen diameter was < 50 um (the equivalent of six red blood cells) and was excluded when the lumen diameter was > 50 um or vessel walls contained muscle. Stained single cells or groups of cells stained with no lumen were regarded as independent micro-vessels. The MVD was determined by counting the number of CD31-positive micro-vessels.

### Statistical analysis

Data were analyzed using the SPSS version 21.0 software (SPSS Inc., Chicago, IL, USA). Categorical data were expressed as ratios and percentages, and differences among groups were compared using the Chi-square test. Continuous data were displayed as means ± standard deviations and differences among multiple groups were analyzed by one-way analysis of variance (ANOVA). Two-tailed *P* < 0.05 was considered statistically significant.
