# Correction: MicroRNA-126 inhibits tumor proliferation and angiogenesis of hepatocellular carcinoma by down-regulating EGFL7 expression

**DOI:** 10.18632/oncotarget.28066

**Published:** 2021-08-31

**Authors:** Ming-Hua Hu, Chen-Yang Ma, Xiao-Ming Wang, Chen-Dong Ye, Guang-Xian Zhang, Lin Chen, Jin-Guo Wang

**Affiliations:** ^1^ Department of Surgery, Yijishan Hospital, Wannan Medical College, Wuhu 241001, P.R. China; ^2^ Department of Surgery, The Second Affiliated Hospital, Wannan Medical College, Wuhu 241001, P.R. China

**This article has been corrected:** The image in Figure 8, panel A is an accidental duplicate of the image in Figure 8, panel E. The corrected Figure 8, obtained using the original data, now shows the proper panel A. The authors declare that these corrections do not change the results or conclusions of this paper.

Original article: Oncotarget. 2016; 7:66922–66934. 66922-66934. https://doi.org/10.18632/oncotarget.11877

**Figure 8:**
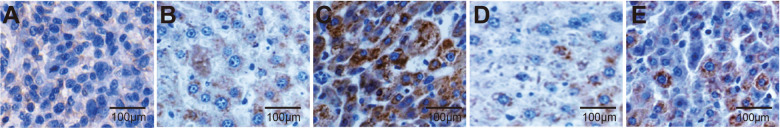
The VEGF-positive rate of transplanted tumors in nude mice among different groups detected by immunohistochemistry (× 200). (**A**) The blank group; (**B**) The miR-126 mimics group; (**C**) The miR-126 inhibitors group; (**D**) The si-EGFL7 group; (**E**) The miR-126 inhibitors + si-EGFL7 group.

